# Prognostic Nutritional Index (PNI) and Controlling Nutritional Status (CONUT) score for predicting outcomes of breast cancer: A systematic review and meta-analysis

**DOI:** 10.12669/pjms.39.5.7781

**Published:** 2023

**Authors:** Puchao Peng, Lijie Chen, Qinjing Shen, Zhouming Xu, Xiufang Ding

**Affiliations:** 1 Puchao Peng, Department of Breast Surgery, Huzhou Maternity & Child Health Care Hospital, Huzhou 313000, Zhejiang Province, P.R. China; 2 Lijie Chen, Department of Breast Surgery, Huzhou Maternity & Child Health Care Hospital, Huzhou 313000, Zhejiang Province, P.R. China; 3 Qinjing Shen, Department of Breast Surgery, Huzhou Maternity & Child Health Care Hospital, Huzhou 313000, Zhejiang Province, P.R. China; 4 Zhouming Xu, Department of Breast Surgery, Huzhou Maternity & Child Health Care Hospital, Huzhou 313000, Zhejiang Province, P.R. China; 5 Xiufang Ding, Department of Breast Surgery, Huzhou Maternity & Child Health Care Hospital, Huzhou 313000, Zhejiang Province, P.R. China

**Keywords:** Breast cancer, Malnutrition, Prognosis, Mortality, Recurrence

## Abstract

**Objective:**

To assess prognostic nutritional index (PNI) and controlling nutritional status (CONUT) score could predict overall survival (OS) and disease-free survival (DFS) in patients with breast cancer.

**Methods:**

PubMed, Embase, ScienceDirect, CENTRAL, and Google Scholar were searched from 1^st^ January 2000 to 10^th^ October 2021 for studies assessing the association between PNI or CONUT and outcomes of breast cancer by following the PRISMA guidelines. Keywords used were “Prognostic nutritional index”, “Controlling nutritional status”, “CONUT”, and “Breast cancer”.

**Results:**

Nine studies were included. On pooled analysis, we noted a statistically significant improved OS in patients with high PNI as compared to low PNI. Meta-analysis revealed no significant difference in DFS between patients with high PNI and low PNI. However, on the exclusion of one study, we noted that high PNI was associated with significantly improved DFS as compared to low PNI. On pooled analysis, we also noted that a high CONUT score was associated with significantly reduced OS in breast cancer patients.

**Conclusion:**

Our results indicate that PNI is an important prognostic factor for patients with breast cancer. Pre-treatment low PNI is associated with worse OS and DFS. Scarce data also indicates that a high CONUT score is predictive of poor OS in breast cancer.

## INTRODUCTION

Breast cancer is the commonest malignancy seen in women across the globe and is the leading cause of female death from cancer.^1^ The incidence of breast cancer continues to rise worldwide contributing to an increased burden on the healthcare system.^2^ Over the past few decades, intense basic and clinical research has led to an improved understanding of the complex pathophysiological mechanism of breast cancer along with significant improvements in surgery, chemotherapy, radiotherapy, targeted therapy as well as immunotherapy.^3^ However, despite the technological advances, overall survival (OS) and disease-free survival (DFS) amongst breast cancer patients remain unpredictable and is a cause of concern. In recent times, several molecular markers have been explored to predict clinical outcomes of breast cancer, however, the time-consuming process and associated high costs have limited their clinical application.^4^which contained 22 stroma samples (15 were from normal breast and 7 were from invasive ductal carcinoma tumor samples Therefore, to guide clinicians and allow personalized treatment programs, there is a need for reliable, easy to use and inexpensive prognostic markers for breast cancer patients.

In recent times, there has been a focus on lifestyle factors like physical activity, diet, smoking, alcohol consumption, and nutritional status on cancer-related mortality.^5^ Indeed, a large proportion of cancer patients suffer from malnutrition either due to direct physiological effects of the malignancy (malabsorption, gastrointestinal obstruction, diarrhea) or due to the body’s tumor response (leading to anorexia and impaired metabolism) or due to adverse effects of anti-cancer therapy.^6^ The presence of malnutrition in cancer patients has been associated with poor response to anti-cancer therapy, increased risk of postoperative complications, and poor OS.^7^

To allow qualification of the nutritional and immune status of the patient, two important biomarkers, namely the prognostic nutritional index (PNI) and the controlling nutritional status (CONUT) score have been developed.^8^,^9^ The PNI, which is calculated by adding the serum albumin and total lymphocyte counts, was initially used to assess the nutritional status of patients undergoing gastrointestinal surgery.^10^ However, it has gradually been recognized as an important prognostic factor for several solid tumors.11-13 Similarly, the CONUT score which is assessed by combining cholesterol, albumin, and lymphocyte counts of the patient has also been reported to predict outcomes of several cancers.^14^-^16^ In the past few years, several systematic reviews and meta-analyses studies have analyzed the prognostic role of PNI and CONUT for a variety of different solid tumors.^11^-^16^

Nevertheless, to the best of our knowledge, no study has been conducted to review evidence on the ability of these markers to predict outcomes of breast cancer. Therefore, our review was designed to assess if PNI and CONUT could predict survival outcomes in patients with breast cancer.

## METHODS

### Database search:

The review was registered on PROSPERO (CRD42021282980). For the purpose of this review, we searched the databases of PubMed, Google Scholar, Embase, CENTRAL, and ScienceDirect for studies reporting the association of PNI/CONUT and survival of breast cancer patients. An English-language only search was conducted from 1^st^ January 2000 to 10^th^ October 2021, using the terms: “Prognostic nutritional index”, “Controlling nutritional status”, “CONUT”, and “Breast cancer” (Supplementary Table-I). The first set of search results were scrutinized by their titles and abstracts and relevant studies were noted. We then read the full texts of the selected studies and matched them against eligibility criteria. Two reviewers conducted the entire exercise and any disagreements were cleared in consultation with the third reviewer. We also conducted a hand-search of the bibliography of included studies to check for any possible exclusions. The entire review was conducted following the PRISMA guidelines.^17^

**Supplementary Table-I:** Search strategy.

Search number	Query	Search Details
1	(Prognostic nutritional index) AND (Breast cancer)	("nutrition assessment"[MeSH Terms] OR ("nutrition"[All Fields] AND "assessment"[All Fields]) OR "nutrition assessment"[All Fields] OR ("prognostic"[All Fields] AND "nutritional"[All Fields] AND "index"[All Fields]) OR "prognostic nutritional index"[All Fields]) AND ("breast neoplasms"[MeSH Terms] OR ("breast"[All Fields] AND "neoplasms"[All Fields]) OR "breast neoplasms"[All Fields] OR ("breast"[All Fields] AND "cancer"[All Fields]) OR "breast cancer"[All Fields])
2	(Controlling nutritional status) AND (breast cancer)	("controling"[All Fields] OR "controllability"[All Fields] OR "controllable"[All Fields] OR "controllably"[All Fields] OR "controller"[All Fields] OR "controller s"[All Fields] OR "controllers"[All Fields] OR "controlling"[All Fields] OR "controls"[All Fields] OR "prevention and control"[MeSH Subheading] OR ("prevention"[All Fields] AND "control"[All Fields]) OR "prevention and control"[All Fields] OR "control"[All Fields] OR "control groups"[MeSH Terms] OR ("control"[All Fields] AND "groups"[All Fields]) OR "control groups"[All Fields]) AND ("nutritional status"[MeSH Terms] OR ("nutritional"[All Fields] AND "status"[All Fields]) OR "nutritional status"[All Fields]) AND ("breast neoplasms"[MeSH Terms] OR ("breast"[All Fields] AND "neoplasms"[All Fields]) OR "breast neoplasms"[All Fields] OR ("breast"[All Fields] AND "cancer"[All Fields]) OR "breast cancer"[All Fields])
3	(CONUT) AND (Breast cancer) - Spellcheck off	"CONUT"[All Fields] AND ("breast neoplasms"[MeSH Terms] OR ("breast"[All Fields] AND "neoplasms"[All Fields]) OR "breast neoplasms"[All Fields] OR ("breast"[All Fields] AND "cancer"[All Fields]) OR "breast cancer"[All Fields])

### Inclusion criteria:

- All types of cohort studies, cross-sectional studies, and case-control studies conducted on patients with breast cancer.
- Studies were to report the association between PNI or CONUT and outcomes of breast cancer.
- Outcomes of interest to this review were OS and DFS which was reported as odds ratios (OR), risk ratios (RR) or Hazard ratios (HR) with 95% confidence intervals (CI).

### Exclusion criteria:

- Studies not reporting outcomes of interest.
- Studies on a mixed cohort of cancer patients not reporting separate data for breast cancer.
- Review articles and case reports.
- Studies with a repeated or overlapping sample. For two studies with overlapping data, the largest study was included.

### Data extraction and Risk of bias assessment:

The following details were extracted by two reviewers: first author name, year, study database, study type, study duration, sample size, mean age, clinical stage, treatment, the index used (PNI or CONUT), the timing of measurement of the index, cut-off value, the method used to determine cut-off, follow-up duration, and outcomes.

Two reviewers judged the study quality using the Newcastle-Ottawa scale (NOS)^18^ which has three domains, namely, study population, comparability, and outcomes. Each of them is awarded stars based on predetermined questions. The maximum score achievable is nine.

### Statistical analysis:

We extracted outcome data for OS and DFS and combined them to compute the total effect size as HR and 95% confidence intervals (CI) in a random-effects model. We assessed inter-study heterogeneity using the I^2^ statistic. I^2^=25-50% meant low, 50-75% meant medium, and more than 75% meant substantial heterogeneity. Due to limited number of studies, funnel plots were not used to assess publication bias. A sensitivity analysis was performed to examine the influence of each study on the review results. Each study was removed one at a time and the pooled effect estimate was recalculated for the remaining studies. The review was conducted using “Review Manager” (RevMan, version 5.3; Nordic Cochrane Centre [Cochrane Collaboration], Copenhagen, Denmark; 2014).

## RESULTS

### Search results and details of included studies:

**Fig.1:**
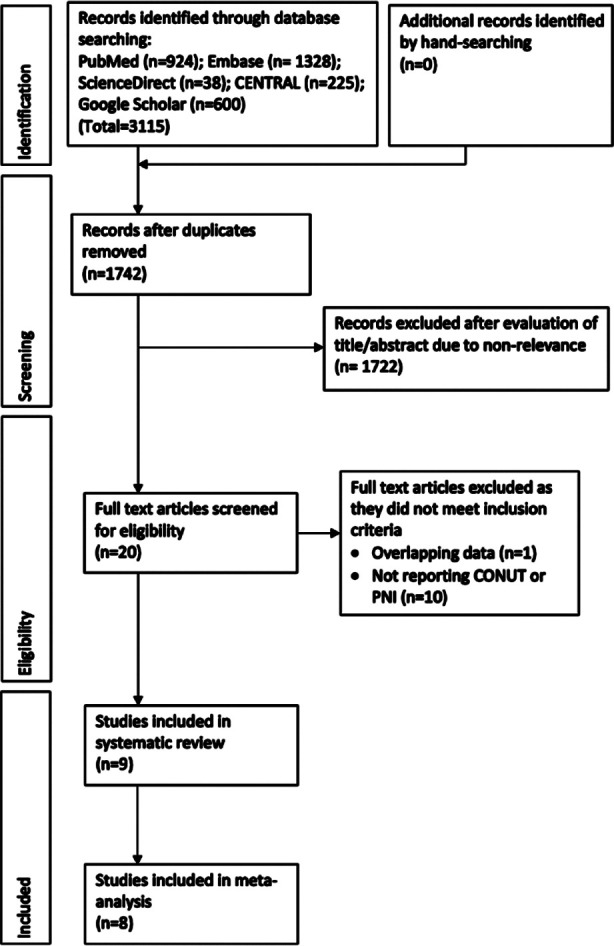
Study flow-chart.

The PRISMA flowchart of the study is presented in Fig.1. Twenty studies were assessed by their full-texts and finally, nine studies^8^,^9^,^19^-^25^ were included in this review (Table-I). All were retrospective cohort studies analysing prior hospital records and published between 2014 to 2021. One study19 was from the Czech Republic while the remaining were from either Japan or China. The sample size of the included studies ranged from 191 to 1367 patients. The clinical stage of cancer varied across studies. In three studies8,^21^,^22^ only surgical patients were included while in another three studies9,^20^,^23^ neoadjuvant therapy was used before surgery. In the remaining studies, breast cancer patients underwent adjuvant therapy after surgery. Out of the nine studies^8^,^9^,^19^-^25^ only two studies^8^,^21^ reported outcomes based on CONUT score while the remaining used PNI. The two studies^8^,^21^ using CONUT used the same cut-off (≥3) but the cut-off of PNI varied across studies. The mean/median follow-up was more than one year for most studies.^8^,^9^,^20^-^25^ One study^19^ had a high risk of bias and scored six points on NOS while the remaining studies^8^,^9^,^20^-^25^ were of moderate risk of bias and scored eight points.

**Table-I:** Details of included studies.

Study	Location	Database	Included period	Sample size	Age (Years)	Clinical stage	Treatment	Index	Timing of measurement	Cut-off value	Cut-off determination	Follow-up	NOS score
Chen 20219	China	National Cancer Center	1998-2016	785	47	I-III	Neoadjuvant chemotherapy and surgery	PNI	7 days prior to neoadjuvant chemotherapy	51	ROC curve	Up to 20 years	8
Hua 202022	China	Sun Yat-sen University Cancer Center	2010-2012	380	47	I-II	Surgery	PNI	Within 3 days of surgery	52	ROC curve	63.1 months (3.2-95.9 months)	8
Huang 20208	China	Sun Yat-sen University Cancer Center	2010-2012	1367	NR	NR	Surgery	CONUT	Within 1 week before surgery	3	Prior research	5.9 years (0.02-8.82 years)	8
Li 202021	China	West China Hospital	2007-2010	1364	NR	I-III	Surgery	CONUT	Within 1 week before surgery	3	ROC curve	61.7 months	8
Melichar 201719	Czech republic	Palacký University Medical School and Teaching Hospital	NR	418	NR	NR	NR	PNI	Before starting treatment	NR	NR	NR	6
Mohri 201624	Japan	Toyama Hospital	2006-2015	212	66	I-III	Surgery with or without adjuvant chemo/endocrine therapy	PNI	Just before surgery	52.8	ROC curve	47.7 months	8
Oba 202020	Japan	Shinshu University Hospital	2005-2016	191	51.2	I-IV	Neoadjuvant chemotherapy and surgery	PNI	Before and after neoadjuvant chemotherapy	53.1	ROC curve	51 months (1-151 months)	8
Wang 201923	China	Renji Hospital	2013-2018	202	NR	NR	Neoadjuvant chemotherapy and surgery	PNI	7 days prior to neoadjuvant chemotherapy	55	Median value	26 months (16-42 months)	8
Yang 201425	China	Tianjin Medical University Cancer Institute and Hospital	2003-2005	382	50	I-III	Surgery with adjuvant chemo/radiotherapy	PNI	Just before surgery	48.7	ROC curve	74 months (6-101 months)	8

ROC: receiver operating characteristic; PNI: prognostic nutritional index; CONUT: Controlling nutritional status; NR: not reported.

### Meta-analysis:

**Fig.2:**
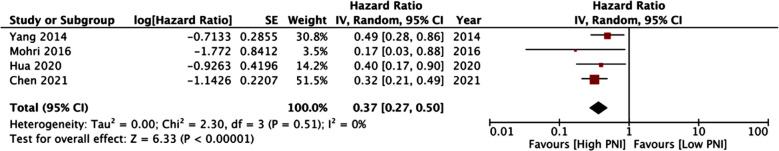
Meta-analysis of PNI scores and overall survival in breast cancer.

Five studies^9^,^19^,^22^,^24^,^25^ reported data on the association between PNI and OS. Of these four studies^9^,^22^,^24^,^25^ compared outcomes using specific cut-offs of PNI which ranged from 48.7 to 52.8, while one study by Melichar et al^19^ used PNI as a continuous variable. Due to this difference, we excluded the study of Melichar et al^19^ from the meta-analysis. On descriptive analysis, Melichar et al^19^ reported no significant relationship between PNI and OS of breast cancer patients (HR: 0.963 95% CI: 0.926-1.002). On pooled analysis of the remaining four studies,^9^,^22^,^24^,^25^ we noted a statistically significant improved OS in patients with high PNI as compared to low PNI (HR: 0.37 95% CI: 0.27, 0.50 I^2^=0% p<0.00001) (Fig.2). On sensitivity analysis, the result did not change on the exclusion of any study.

**Fig.3:**
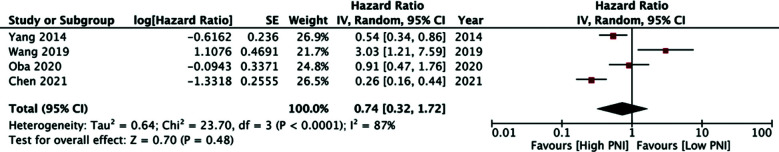
Meta-analysis of PNI scores and disease-free survival in breast cancer.

Four studies^9^,^20^,^23^,^25^an indicator of nutritional and immunological status, has an impact on the long-term outcomes in triple-negative breast cancer (TNBC reported DFS based on PNI. Meta-analysis revealed that there is no significant difference in DFS between patients with high PNI and low PNI (HR: 0.74 95% CI: 0.32, 1.72 I^2^=87% p=0.48) (Fig.3). However, on the exclusion of the study of Wang et al^23^ from the meta-analysis, we noted that high PNI was associated with significantly improved DFS as compared to low PNI (HR: 0.49 95% CI: 0.25, 0.96 I^2^=78% p=0.04).

**Fig.4:**
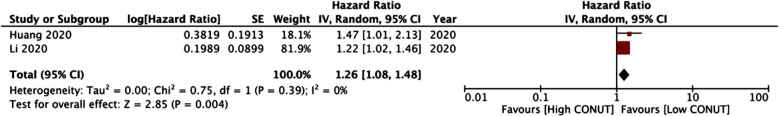
Meta-analysis of CONUT scores and overall survival in breast cancer.

Two studies^8^,^21^ reported data on the prognostic impact of CONUT on OS of breast cancer patients. On pooled analysis, a high CONUT score was associated with significantly reduced OS (HR: 1.26 95% CI: 1.08, 1.48 I^2^=0% p=0.004) (Fig.4). The association between CONUT and DFS for breast cancer patients was reported only by Huang et al.^8^ The authors reported significantly reduced DFS in patients with high CONUT scores vs low CONUT scores (HR: 2.104 95% CI: 1.172-3.779).

## DISCUSSION

Our systematic review and meta-analysis is the first to explore the association between PNI and CONUT scores and outcomes of breast cancer patients. Our results indicate that patients with high PNI scores, measured before starting therapy, have better overall survival as compared to patients with low PNI. PNI does not seem to impact DFS, however, the results were consistent on sensitivity analysis. Secondly, scarce data also indicates that patients with high CONUT scores have significantly reduced OS as compared to patients with low CONUT scores.

The role of PNI in predicting prognosis has received significant attention in the past decade. The prognostic significance of PNI has not only been validated in several cancer phenotypes but also for non-cancerous pathologies. Hayashi et al^26^ in a recent study on 453 patients undergoing cardiovascular surgery have demonstrated that low PNI significantly increases the risk of postoperative complications and reduces survival. Candeloro et al.^27^ have also noted significantly increased short-term and long-term mortality with low PNI in a cohort of elderly patients hospitalized for acute decompensated heart failure. Zhang et al^28^ have shown that low PNI is associated with worse outcomes in pediatric patients with renal dysfunction. Kim et al^29^ have noted an increased risk of complications and poor survival amongst low PNI patients undergoing lung transplantation. Similarly, several researchers have assessed the prognostic significance of PNI for different cancers^11^-^13^ but to date, no meta-analysis has assessed the evidence on the association between PNI and breast cancer.

In our review, we pooled data from six studies^9^,^20^,^22^-^25^ analyzing the relationship between pre-treatment PNI and outcomes of breast cancer. Our analysis revealed that patients with low PNI have a poor OS with a 63% higher risk of mortality vs those with high PNI. On the other hand, we noted PNI did not predict DFS amongst breast cancer patients. However, these results must be interpreted in light of the sensitivity analysis. No change in the significance of outcomes on sensitivity analysis of OS with 0% heterogeneity provides robustness to our conclusions. It presents high-quality evidence that pre-treatment PNI is an important prognostic indicator for breast cancer patients. Our results concur with studies reporting the prognostic significance of PNI for other malignancies. Wang et al^13^ in a meta-analysis of 21 studies have demonstrated low PNI to be associated with poor OS as well as DFS in lung cancer patients. Tu et al^11^ in a recent meta-analysis of 10 studies noted that nasopharyngeal carcinoma patients with low pretreatment PNI had significantly poor OS, DFS, distant metastasis-free survival, and locoregional recurrence-free survival as compared to high PNI patients. In another meta-analysis, Li et al^12^ have found significantly poor OS with low PNI amongst pancreatic cancer patients. Similar results have been replicated in patients with gastric cancer,^30^ head and neck cancer,31 and renal cancer^32^ as well. An important difference between these prior results and our review is that we noted no difference in DFS based on PNI. However, on the exclusion of the study of Wang et al^23^ the results did demonstrate significantly worse DFS with low PNI. Such variation could be explained by the patient selection in the study of Wang et al.^23^ The authors in their study defined high PNI as a score of ≥55 while low PNI ranged from 45-55. Therefore, their comparison was between patients with excessively high PNI vs high PNI rather than high PNI vs low PNI. Increased risk of mortality with high PNI in their study is indicative of a U-shaped relationship between PNI and outcome with low-PNI (as defined by other studies) and excessively high-PNI resulting in worse outcomes.^23^ Such association has been noted between body mass index and all-cause cancer mortality.^33^

The reason for poor outcomes with low PNI has several explanations. Since PNI is measured by serum albumin and lymphocyte counts, low PNI could be indicative of hypoalbuminemia which is reflective of the nutritional status of the patient.^23^ Malnutrition is known to impact host immunity and therefore cancer outcomes.^34^ Lymphocyte counts are reflective of cell-mediated immunity which is an essential component of cancer defense. High lymphocyte counts are known to improve OS in breast cancer patients irrespective of clinical and pathological characteristics.^35^ These factors could contribute to poor outcomes with low PNI in breast cancer patients.

In the second part of our meta-analysis, we explored the relationship between CONUT and outcomes of breast cancer. However, our review was limited by the scarce data available in the literature. The difference between CONUT and PNI is that the former includes cholesterol levels in addition to serum albumin and lymphocyte counts.^21^ Cholesterol has an important role in cell membrane formation and immunity which enables immunocompetent cells to launch an immune response against cancer cells.^36^ Furthermore, cholesterol levels have also been linked to tumorigenesis.^37^ Therefore, as compared to PNI, CONUT may be a better biological marker as it measures systemic inflammation as well as the nutritional and immunological state of the patient.^21^ Indeed, several studies have demonstrated that high CONUT scores are associated with poor outcomes in patients with cancer.^14^-^16^ Our results concur with these prior studies^14^-^16^ as we also noted significantly reduced OS with high CONUT scores in patients with breast cancer. It is important to note that CONUT scores are divided based on the degree of malnutrition detected as normal (0-1), light (2-4), moderate (5-8), and severe (9-12).^21^ Hence, high CONUT values indicate poor nutritional status which is opposite to that of PNI scores.

### Limitations:

Firstly, the number of studies available for inclusion in the review was not high. The number of studies reporting data on the same outcome was further small which may have reduced the statistical power of our analysis. Secondly, all included studies were retrospective cohort in nature and the risk of selection bias cannot be ruled out. Thirdly, the baseline clinicopathological stage of breast cancer varied across the included studies.

Since most studies included a mix of patients with different cancer stages and due to a limited number of included studies, we were unable to perform a subgroup analysis for the same. Furthermore, the treatment protocols were also different across studies. Some patients received neoadjuvant therapy while others received adjuvant treatments and we could not explore how do these variations impact the association between PNI/CONUT and patient outcomes. Also, the cut-off of PNI was not the same across included studies and this may have skewed outcomes. Finally, most studies in our review were from China and Japan and therefore the findings cannot be generalized to the global population.

Nevertheless, our study is novel as it is the first systematic review to assess if PNI and CONUT could predict outcomes of breast cancer. We pooled only adjusted data from the included studies and this may have partially offset the impact of other confounders on the review outcomes.

## CONCLUSIONS

Our results indicate that PNI is an important prognostic factor for patients with breast cancer. Pre-treatment low PNI is associated with worse OS and DFS. Scarce data also indicates that a high CONUT score is predictive of poor OS in breast cancer. There is a need for further studies assessing the relationship between PNI/CONUT and breast cancer outcomes while taking into account baseline clinical stage and treatment protocols to further strengthen the evidence.

### Authors’ contributions:

**PP:** Conceived and designed the study.

**LC, QS, ZX and XD:** Collected the data and performed the analysis.

**PP:** Was involved in the writing of the manuscript and is responsible for the integrity of the study.

All authors have read and approved the final manuscript.
